# Using Acute Flaccid Paralysis Surveillance as a Platform for Vaccine-Preventable Disease Surveillance

**DOI:** 10.1093/infdis/jiw593

**Published:** 2017-07-01

**Authors:** Steven G. F. Wassilak, Cheryl L. Williams, Christopher S. Murrill, Benjamin A. Dahl, Chima Ohuabunwo, Rudolf H. Tangermann

**Affiliations:** 1 Global Immunization Division, Center for Global Health, Centers for Disease Control and Prevention, Atlanta, Georgia; and; 2 Polio Eradication Department, World Health Organization, Geneva, Switzerland

**Keywords:** poliovirus, AFP surveillance, vaccine preventable disease surveillance, polio transition planning, integrated disease surveillance.

## Abstract

Surveillance for acute flaccid paralysis (AFP) is a fundamental cornerstone of the global polio eradication initiative (GPEI). Active surveillance (with visits to health facilities) is a critical strategy of AFP surveillance systems for highly sensitive and timely detection of cases. Because of the extensive resources devoted to AFP surveillance, multiple opportunities exist for additional diseases to be added using GPEI assets, particularly because there is generally 1 district officer responsible for all disease surveillance. For this reason, integrated surveillance has become a standard practice in many countries, ranging from adding surveillance for measles and rubella to integrated disease surveillance for outbreak-prone diseases (integrated disease surveillance and response). This report outlines the current level of disease surveillance integration in 3 countries (Nepal, India, and Nigeria) and proposes that resources continue for long-term maintenance in resource-poor countries of AFP surveillance as a platform for surveillance of vaccine-preventable diseases and other outbreak-prone diseases.

The Global Polio Eradication Initiative (GPEI) is close to achieving the long-sought eradication goal, with ongoing transmission of wild poliovirus type 1 (WPV1) being restricted to limited areas of just 3 countries, Nigeria, Afghanistan, and Pakistan. In August 2016, indigenous WPV1 was again detected in Nigeria, following an absence of reported cases since July 2014, which had resulted in removing Nigeria from the World Health Organization’s (WHO’s) list of endemic countries. The Polio Eradication and Endgame Strategic Plan 2013–2018 highlights the importance of collaboration within the global health community to ensure that investments made for the global eradication of polio will allow the transition of polio eradication assets to contribute to other future health goals [1]. A principal component of polio transition planning is to maintain and mainstream essential polio functions—including surveillance and outbreak response—as ongoing public health functions. Knowledge and lessons learned from polio eradication activities will be shared, and polio program capacities, resources, and infrastructure will be transitioned to support other public health priorities, particularly in order to strengthen surveillance for other vaccine-preventable diseases (VPDs) through the integration of disease surveillance activities.

Surveillance for acute flaccid paralysis (AFP) is a fundamental cornerstone of the GPEI. Pioneered in the Americas during the late 1980s [2, 3], AFP surveillance is conducted in 194 member states of the WHO. Coupled with a laboratory network and data management system, surveillance for AFP allows for detection of cases of polio disease wherever poliovirus may still be circulating. AFP surveillance will also provide evidence on the absence of poliovirus transmission when, in the presence of high-quality AFP surveillance systems, no poliovirus is isolated. The quality of AFP surveillance is evaluated by tracking 2 principal performance indicators. The nonpolio AFP rate (ie, the number of AFP cases not due to polio per 100000 children aged <15 years per year) is indicative of the sensitivity of the surveillance system; a nonpolio AFP rate ≥ 2/100000 is considered sufficiently sensitive to identify wild poliovirus (WPV) or circulating vaccine-derived poliovirus (cVDPV) cases where poliovirus continues to circulate [4]. The second performance indicator is the proportion of AFP cases for which adequate stool specimens were collected; the target is ≥80%, indicating surveillance can effectively identify poliovirus among children with AFP [4]. Specimens are considered adequate if 2 stool specimens are collected within 14 days of paralysis onset, at least 24 hours apart, arriving at a WHO-accredited polio laboratory in “good” condition^a^ (reverse cold chain maintained and received without leakage or desiccation at a WHO–accredited laboratory. Reverse cold chain is maintained when stool specimens are stored immediately after collection at 4–8°C (32–39°F), frozen at −20°C (−4°F) when received for processing, and shipped to a WHO–accredited laboratory in dry ice or cold packs. Freezing of specimens is unnecessary if specimens can be received at a WHO–accredited laboratory within 72 hours of collection). However, meeting the 2 key AFP performance indicators alone does not yet assure high surveillance quality; high quality can only be assured if surveillance activities are well supervised.

This report outlines the current level of integration of AFP surveillance with surveillance for other diseases in 3 countries—Nepal, India, and Nigeria—and suggests how surveillance for VPDs and other outbreak-prone diseases can benefit in the long-term from maintaining AFP surveillance. In addition, this report underscores the importance of strengthening and maintaining the existing surveillance infrastructure for a number of years following both certification of polio eradication and suspension of bivalent oral polio vaccine (bOPV) use, in order to demonstrate the continued absence of poliovirus transmission, and to rapidly identify and respond to any reemergence or importations of poliovirus. This is critical to monitoring and maintaining a polio-free world, and to protecting the significant investment made towards global polio eradication.

## HISTORICAL OVERVIEW

The GPEI has developed and maintained AFP surveillance as the primary means of monitoring the impact of polio eradication activities, enabling prompt poliovirus detection and outbreak response throughout the world. Building national AFP surveillance capacity (including investigation and documentation of AFP cases, community investigation and search for additional cases, transport of specimens to the laboratory under reverse cold chain conditions, as well as rapid data handling and linkage with laboratory results) has allowed the training and creation of a well-versed surveillance workforce able to respond to other communicable disease emergencies, such as cholera and meningitis [5]. Active surveillance, with regular visits of trained public health staff to search for cases at health facilities, is a critical strategy for AFP surveillance systems to ensure highly sensitive and timely AFP case detection. Active surveillance visits are conducted at facilities that are likely to see not only cases of AFP, but also other vaccine-preventable and outbreak-prone diseases. In many countries, active surveillance visits are already used to detect and report other conditions in addition to AFP. The majority of WHO member states still devote considerable human and financial resources to maintain and improve effective AFP surveillance systems, creating multiple opportunities for adding additional surveillance components using GPEI assets; this is facilitated by the fact that there is generally only 1 district officer responsible for all disease surveillance. Provided that resources for active AFP surveillance continue to be available, integrated surveillance can become standard procedure, ranging from simply adding surveillance for measles and rubella to a completely integrated disease surveillance system for outbreak-prone diseases (integrated disease surveillance and response [IDSR]).

According to a survey of staff in the WHO African Region office and personnel in ministries of health of member states in the region conducted in 2000, 26 (81%) of the 32 countries who participated reported using AFP surveillance resources for the surveillance of other infectious diseases, including measles, neonatal tetanus, cholera, meningitis, and yellow fever [5]. As of the end of 2003, 131 (66%) of 198 countries globally had adapted their AFP surveillance systems for surveillance of measles and other VPDs, such as yellow fever [6]. This trend has continued so that currently, the majority of countries are conducting AFP surveillance in conjunction with at least some other VPDs. Surveillance for additional VPDs has been added, depending on a country’s immunization schedule and disease burden, including surveillance for meningitis and encephalitis to track Japanese encephalitis and bacterial meningitis [7].

In regions certified as polio free for many years (the Americas, Europe, and the Western Pacific Region), the sensitivity and quality of AFP reporting has decreased somewhat over time [8, 9]. The main reason for this is that health workers are aware that polio has been eliminated for many years, and may no longer see the need for continued vigilance; additionally, resources available for surveillance have decreased over time, followed by a decrease in the frequency and quality of active surveillance visits to health facilities.

What is proposed here is that the level of support for surveillance of VPDs and other outbreak-prone diseases be maintained by a consortium of interested parties, to sustain a vigorous capacity for outbreak detection and response and for tracking the progress of accelerated disease control (particularly in the most resource-poor countries and areas). It is in the long-term interest of both the polio eradication program and countries to transition AFP surveillance into an integrated disease surveillance system. The detection of poliovirus transmission will need to continue for the foreseeable future, beyond the certification of WPV eradication and global withdrawal of bOPV, regardless of the possible source of poliovirus—vaccine manufacturer, intentional or unintentional release from laboratory stocks, or vaccine-derived poliovirus shed by a person with primary immunodeficiency—or type of virus (wild, vaccine-derived, or Sabin-related poliovirus), to ensure swift identification of and response to re-emergent polioviruses.

Polio eradication transition planning will require each country to consider the extent to which polio assets are already integrated into existing health programs. The current functional integration of AFP surveillance with surveillance for other VPDs is discussed for Nepal, India, and (in more depth) for Nigeria.

### Nepal

Nepal provides an example of the benefits of utilizing polio program expertise for other health programs, but also demonstrates how integration of polio resources into other health priorities may leave some critical functions “at risk” without polio funding. The polio-funded Program for Immunization Preventable Disease (IPD), a separate WHO program supporting the National Immunization Program (NIP), is the backbone of surveillance activities in Nepal. Begun in 1998 and originally focused on AFP surveillance, the IPD was expanded in 2003 to include tracking of other VPDs (which are still monitored today), including suspected measles and rubella cases, acute encephalitis syndrome for Japanese encephalitis, and neonatal tetanus, as well as technical support to the routine immunization program. The program uses both passive reporting and active monitoring and case investigation. Nepal was able to benefit from the AFP program’s existing infrastructure and expertise on how to conduct quality surveillance, but without continued funding for the IPD, it is likely the program, and thus, surveillance of VPDs in Nepal, would significantly degrade [10, 11].

### India

WHO Country Office for India has already taken concrete steps to manage polio-funded resources in a post-eradication environment and to transition the capacities, processes, and assets created by the polio program to support other VPD surveillance and strengthen health systems. Since 1997, a large workforce of Indian national surveillance officers, the National Polio Surveillance Project (NPSP), has been employed by WHO in a collaboration with the government of India. Ever since the last case of WPV was observed in India in 2011, the roles of the approximately 300 NPSP surveillance medical officers (SMOs) have changed. For hundreds of NPSP workers, the workload associated with AFP surveillance and with planning and supervising polio supplementary immunization activities has gradually shifted from WHO to government medical officers, enabling these SMOs to take on additional diseases surveillance functions. For example, the percentage of AFP cases investigated by government officers, as opposed to WHO surveillance officers, increased from 35% in 2009 to 79% in 2014 [12]. The process of selecting and hiring NPSP medical officers and field volunteers has changed considerably, with the hiring of field monitors now outsourced to a specialized agency, and an increasing number of staff selected and employed directly by the government. Training and capacity building have been provided to help NPSP SMOs adjust to new roles supporting routine immunization and surveillance for other VPDs, including measles and rubella. Additionally, NPSP training and program monitoring have improved cold chain handling capabilities, which were critical to the introduction of new vaccines such as hepatitis B and Japanese encephalitis vaccines to the Universal Immunization Program [11–13].

### Nigeria

Nigeria serves as an example of the potential for establishing integrated disease surveillance, building on AFP surveillance, but it also illustrates the inherent limitations. In other countries of the WHO African Region, there have been smaller investments and similar attempts at integration.

Nigeria’s governmental administration is divided into 36 states with 1 Federal Capital Territory, and 774 districts (or Local Government Areas [LGAs]). Each LGA has a disease surveillance and notification officer (DSNO) who has been trained to detect and investigate AFP cases, and to detect outbreaks of suspected measles, meningitis, and cholera. The DSNO is trained to visit and conduct active surveillance at key health facilities, and to maintain contact with a network of community informants, including community leaders, patent medicine sellers, traditional birth attendants, and traditional healers, to enhance the detection of AFP. Each health facility also has an AFP focal person who is expected to report to the LGA DSNO on the presence or absence of AFP during a given week. Ideally, supportive supervision is conducted to evaluate completeness and timeliness of visits. Record logs of patient visits at health facilities are reviewed to assess whether the focal person responsible for collecting weekly information on the occurrence of AFP has been properly informed of new cases, and that the reporting has been conducted correctly. The DSNO instructs parents of a child meeting the WHO case definition of AFP (as verified by WHO staff) on the collection of 2 stool specimens using standard specimen containers. He or she retrieves the specimens, once available, and is responsible for organizing the prompt and safe specimen transport to 1 of 2 national laboratories, under appropriate reverse cold chain conditions. In addition to verifying the AFP cases, WHO staff track the timely transportation of specimens and monitor the AFP surveillance performance indicators for each LGA. Following the successful transportation of specimens to the laboratory, the DSNO (or his/her designee) is reimbursed for expenses, an important tool to motivate the DSNO to continue to perform reliable AFP surveillance fieldwork. In addition, there are monthly state-based meetings held to discuss AFP surveillance and immunization topics that are attended by the LGA DSNOs; the modest per diem reimbursements for these meetings provide further incentive to the field surveillance workers. The LGA administration has the responsibility to pay the LGA DSNOs’ salaries and should also provide transport allowance for surveillance activities; however, most of the expenses associated with AFP surveillance are borne by WHO, with little or no available government funding for surveillance work. In addition to the incentives mentioned earlier, WHO also provides modest monthly transport allowances for all LGA DSNOs in the country, and also makes petty cash available for other expenses, such as a monthly photocopying petty-cash allowance for focal persons at the health facilities. On the other hand, the relevant state-level surveillance officers in nearly all states are not able to fully participate in supervision of surveillance and of active surveillance for AFP, because of a shortage of funds for transportation. For almost 10 years, DSNOs have been instructed to look for suspect measles cases and collect blood specimens from individual suspected cases or from a small proportion of a cluster of cases. However, unlike for AFP specimens, there is no reimbursement scheme for the collection and transport of blood specimens for measles confirmation, which could present challenges to the operation of measles surveillance. As part of the DSNO’s work responsibilities, weekly forms for outbreak-prone diseases are also routinely received and investigated when reported.

Nigeria used AFP surveillance resources and approaches to facilitate its successful response to the Ebola epidemic in West Africa in 2014. Following the creation of an Ebola Incident Management center that eventually became the national Ebola Emergency Operations Center (E-EOC), surveillance teams using the AFP infrastructure and staff, complemented by graduates and residents of the Nigeria Field Epidemiology Training Program, identified 894 Ebola case contacts, and completed nearly 19000 contact tracing visits. As a result of contact tracing and other measures (including social mobilization and establishment of an Ebola treatment unit), transmission from at least 20 cases of Ebola virus disease was halted, preventing a massive outbreak in the most populous country in Africa [14]. This serves as an excellent example of how polio infrastructure can be leveraged to support other critical disease surveillance and response activities.

## FUTURE OPPORTUNITIES

During more than 25 years of existence, the GPEI, one of the largest ever global health initiatives, has mobilized and trained millions of volunteers, social mobilizers, health workers, technical managers, and leaders, and established a standardized, real-time global surveillance and response capacity [15]. This large poliovirus surveillance infrastructure, established at the country level for routine AFP surveillance, includes highly skilled surveillance staff, communication systems, cold chain capacity and materials, and networks for stool specimen transportation, storage and laboratory testing. The opportunity exists—and has been demonstrated in numerous countries—to use and adapt the same infrastructure, best practices, and platform for the surveillance of other VPDs. Reliable and sensitive surveillance is critical for any VPD, in order to establish estimates of disease burden, contribute to the rapid detection and control of outbreaks, and monitor progress toward control and elimination goals, including assessment of the short- and long-term effectiveness of immunization programs. Surveillance for VPDs includes routine reporting of suspected and confirmed cases of measles, rubella, diphtheria, pertussis, neonatal tetanus, bacterial meningitis, acute viral hepatitis, and other diseases. A core component of a well-functioning VPD surveillance system is the availability of a strong laboratory network to test specimens and to confirm suspected cases initially reported by health facilities. The resources of this lab infrastructure can be integrated with other immunization programs. When 1 VPD lab network (such as the global polio lab network [GPLN]) has been established and is functioning, this greatly facilitates the creation of similar networks for other VPDs.

AFP surveillance infrastructure can also be used to support other health and development priorities [15], which are consistently in need of improved surveillance. Experienced AFP surveillance medical officers can help build integrated surveillance systems. There may be more opportunities for effective handover of capacity and knowledge, such as data quality and effectiveness/monitoring, in addition to tangible assets such as supply chain and labs. While transition planning often focuses on personnel, knowledge from the GPEI program can be transitioned to other programs, such as methods and systems for collecting and reporting, data procedures for using those data for decision-making, and lessons on establishing and operating an accredited global laboratory network and using standards to ensure lab quality.

Collaboration among polio partners and governments has created a model that may inform other health programs. For example, the global measles and rubella laboratory network (GMRLN) was developed based on the successful model of the GPLN. As of 2016, 703 GMRLN laboratories have been established in 191 countries. In addition to testing specimens for measles and rubella, many of these laboratories are also responsible for laboratory-based surveillance of other VPDs in their countries, including yellow fever, Japanese encephalitis, and rotavirus [16]. Laboratories selected to support VPD surveillance should have well-trained staff, the ability to process and test both viral and bacterial specimens, quality assurance and proficiency testing capacity, and information systems for real-time reporting of results. The GPLN and these expanded lab networks will serve not only to continue polio surveillance, but will strengthen other VPD surveillance. The value of these global laboratory networks for polio eradication and future disease prevention and control initiatives is described elsewhere [17].

Continued integration of AFP surveillance systems into national and global disease detection and response programs will be essential to maintaining a polio-free world and strengthening VPD surveillance systems, with the goal of reducing and eliminating other VPDs. It will be critical to have programs that support regional elimination of measles and rubella transmission in the near- to mid-term future, with reliable measles-rubella surveillance systems and lab networks to monitor progress toward these goals. Identifying best practices and lessons learned from field implementation will be an important step in integrating existing AFP surveillance systems with VPD surveillance programs, requiring GPEI partner agencies to conduct careful resource planning in conjunction with government partners and stakeholders. In this way, AFP surveillance can be leveraged for measles/rubella surveillance and control and, ultimately, elimination.

Integration of existing AFP surveillance platforms with other global health initiatives will also be an important component for maintaining and mainstreaming AFP surveillance functions. One such initiative, the Global Health Security Agenda (GHSA) pursues a multilateral and multisectoral approach to strengthen both the global capacity and nations’ capacities to prevent, detect, and respond to human and animal infectious diseases threats [18], in order to achieve the goals of the International Health Regulations (IHR) (2005), a framework for the containment of global public health risks (to which all WHO member states are committed). GHSA is composed of 11 lines of effort (so-called “action packages”) in support of tangible, measurable steps required to prevent outbreaks, detect threats in real time, and rapidly respond to infectious disease threats. Two of these 11 action packages are relevant to this discussion, “Real-Time Surveillance and Reporting” and “Immunization”; both highlight the critical importance of strong disease surveillance (including VPD surveillance) to the success of GHSA. GHSA strives to strengthen national surveillance systems that are able to detect events of significance for public health. By 2012, fewer than 20% of countries were prepared to respond to health threats, as indicated by having met IHR goals. By 2014, about 30% of countries were fully prepared to detect and respond to an outbreak [19]. Collectively, member countries need to improve their capacity to detect and respond to health threats; the standardized, real-time global surveillance and response infrastructure established by GPEI, the largest in the world, has a great capacity to do that. Existing AFP surveillance systems, with their unequaled global reach and reporting efficiency, serve an important role as a foundation for strengthening disease surveillance necessary for global health security and IHR compliance. In turn, the collaborative, capacity-building efforts facilitated by GHSA in support of these goals can serve as a framework to support the maintenance of AFP surveillance systems and facilitate their integration with other VPD surveillance functions.

## CONCLUSIONS

High-quality disease surveillance is essential to detect and respond to outbreaks and to measure the impact of health programs. Trained and experienced AFP surveillance medical officers can use the assets of the AFP surveillance system to help build integrated disease surveillance systems, providing opportunities to strengthen overall immunization programs. This is particularly important for childhood immunization programs in low-income countries. In a closer look at selected countries, it becomes apparent that polio assets are not just helping build capacity for nonpolio-related programs, but are providing the actual capacity for these efforts; in some cases, polio assets are the only resource supporting functions in the country. For these reasons, in resource-poor countries, international partners and country governments must collaborate to support key surveillance functions. Without such a transition, both polio eradication and control and elimination of other VPDs will suffer.

## Notes

***Financial support.*** This work was supported by the US Centers for Disease Control and Prevention (CDC) and the World Health Organization (WHO).

***Supplement sponsorship.*** This work is part of a supplement coordinated by the Task Force for Global Health with funding provided by The Bill and Melinda Gates Foundation and the Centers for Disease Control and Prevention.

***Potential conflicts of interest.*** All authors: No reported conflicts. All authors have submitted the ICMJE Form for Disclosure of Potential Conflicts of Interest. Conflicts that the editors consider relevant to the content of the manuscript have been disclosed.

## Notes

a Reverse cold chain maintained and received without leakage or desiccation at a WHO–accredited laboratory. Reverse cold chain is maintained when stool specimens are stored immediately after collection at 4–8°C (32–39°F), frozen at −20°C (−4°F) when received for processing, and shipped to a WHO–accredited laboratory in dry ice or cold packs. Freezing of specimens is unnecessary if specimens can be received at a WHO–accredited laboratory within 72 hours of collection.
